# Effects of feed form and particle size on growth performance, nutrient digestibility, carcass characteristics, and gastric health in growing-finishing pigs

**DOI:** 10.5713/ab.20.0777

**Published:** 2021-01-14

**Authors:** Yun Yeong Jo, Myung Jae Choi, Woo Lim Chung, Jin Su Hong, Jong Seon Lim, Yoo Yong Kim

**Affiliations:** 1 Feed Innovation Center, Sunjin Company Limited, Seoul 05372, Korea; 2 Research Institute of Agriculture and Life Sciences, Seoul National University, Seoul 08826, Korea

**Keywords:** Feed Processing, Pellet Diet, Particle Size, Growth Performance, Nutrient Digestibility, Gastric Health

## Abstract

**Objective:**

This study was conducted to evaluate the effects of feed processing and particle size on growth performance, nutrient digestibility, carcass characteristics, and gastric health in growing-finishing pigs.

**Methods:**

A total of 360 growing pigs (22.64±0.014 kg initial body weight [BW]) were allocated to 1 of 6 treatments with 6 replicates by BW and sex, and 10 pigs were housed in one pen in a randomized complete block design. The BW and feed intake were recorded to calculate growth performance. For the digestibility trial, a total of 24 barrows with an initial BW of 33.65±0.372 kg were split into 6 treatments with a completely randomized design. Dietary treatments were designed by a 2×3 factorial arrangement of treatments based on two main factors, particle size (600, 750, 900 μm) and feed form (mash and pellet) of diet. Experimental diets were formulated to contain the requirements of the NRC (2012).

**Results:**

The BW and average daily gain were not changed by dietary treatments, and the feed intake of finishing pigs (wks 6 to 12) was increased when the pigs were fed a mash diet (p<0.05). For the overall period, the feed efficiency of pigs was improved with the pellet diet (p<0.01) and reduced particle size (p<0.05). The pellet diet had effects on increasing crude fat digestibility (p<0.01) relative to a mash diet, but there was no considerable change in dry matter and crude protein digestibilities by dietary treatments. In the evaluation of gastric health, a trend for an increased incidence of keratinization in the esophageal region was observed as particle size decreased (p = 0.07).

**Conclusion:**

Feed efficiency could be improved by pellet diet and reduced particle size. Nutrient digestibility, carcass characteristics, and gastric health were not affected by feed form, and particle size ranged from 600 to 900 μm.

## INTRODUCTION

The physical properties of the diet are important factors for determining animal performance, and optimal particle size has been a popular research topic based on this background [1,2]. Reduced particle size may increase the surface area for enzyme digestion, and there are many results describing the positive effects of reduced particle size on the nutrient digestibility of pigs [3,4]. Apparent total tract digestibility (ATTD) of starch was improved with decreased particle size from 920 to 580 μm [5], and reduced particle size had effects on increasing the ATTD of gross energy and crude protein (CP) [6]. Although reduced particle size has many positive effects, it may lead to increased production cost and decreased feed productivity. Furthermore, the responses of different particle sizes were inconsistent in several studies because of different feed intakes [3], altered incidences of gastric ulcers [7,8], and various environmental conditions [9]. Therefore, a study evaluating the optimal particle size is clearly needed to improve the growth of pigs with acceptable production costs.

Pelleting a corn-soybean meal diet had positive effects on improving growth performance, nutrient digestibility, and feed efficiency [10,11]. Ulens et al [12] demonstrated that the feed efficiency of pigs fed a pellet diet was increased relative to those fed a mash diet, and Steidinger et al [13] found that pelleting feed ingredients improved the feed intake of weaning pigs. The major reason for these responses was improved gelatinization of the starch fraction in feed ingredients [10]. Although there were many findings associated with the effects of pellet diet on pigs, limited information is available for the interaction between feed form and particle size of feed ingredients.

Feed form and particle size of diet are dominant factors for determining production cost, but it is difficult to implement optimal standard, because of inconsistent results from previous studies. In this case, the accumulation of meaningful researches and the revalidation process are important to find a conclusion. Thus, this study was conducted to determine the effects of feed processing and particle size on growth performance, nutrient digestibility, carcass characteristics, and gastric health in growing-finishing pigs.

## MATERIALS AND METHODS

### Animal care

All experiments with animals were conducted based on the standard of the Institutional Animal Care and Use Committee provided by Seoul National University (SNUIACUC; SNU-171203-03).

### Animals, housing, and diets

A total of 360 growing pigs ([Yorkshire×Landrace]×Duroc; 22.64±0.014 kg initial body weight [BW]) were used for a 12-wk growth trial and allotted to each of 6 treatments in 6 replicates based on BW and sex in a randomized complete block design. Each pen had 10 pigs and was equipped with half-slotted concrete floors (1.60×3.00 m), water nipples, and a feeder to provide water and feed with *ad libitum* access. Room temperature was controlled stably at 24°C for the 6-wk growing period and 22°C for the 6-wk finishing period. The BW and feed intake were recorded at the 0, 3rd, 6th, 10th, and 12th wks to calculate the average daily gain (ADG), average daily feed intake (ADFI) and gain-to-feed ratio (G/F ratio).

Dietary treatments were designed by a 2×3 factorial arrange ment of treatments, and the main factors were particle size (600, 750, 900 μm) and feed form (mash and pellet) of the diet. Corn and soybean meal were major ingredients in the experimental diets. Grower diets contained 3,300 kcal of metabolizable energy (ME)/kg, 15.00% CP, 1.11% total lysine, 0.66% total Ca, and 0.56% total P, and finisher diets contained 3,275 kcal of ME/kg, 14.00% CP, 1.01% total lysine, 0.52% Ca, and 0.47% total P, respectively. All other nutrients met or exceeded the requirements of NRC (2012) [14], and the formula and chemical composition of the experimental diets are presented in Table 1.

### Production conditions for the experimental diet

The experimental diet was ground by a hammer mill (ANDRITZ Feed & Biofuel, Denmark) equipped with screen sizes of 3.6, 2.6, and 1.6 mm. The average production volume was 4 tons, and the hammer mill screen was changed to control the particle size. The pelleted diets were produced by a 400-horsepower pellet mill (7730-8, CPM, Denmark, 80-mm-thick die with 4.2-mm-diameter holes), and steam was used for conditioning the diets to 75°C before the pelleting process. The temperature of pellet processing was not exceed 80°C with 6.5 to 7.8 kWh for 60 minutes.

### Digestibility trial

For evaluating total tract digestibility, a total of 24 barrows ([Yorkshire×Landrace]×Duroc; 33.65±0.372 kg initial BW) were split into 6 treatments with a completely randomized design. Diets were fed to pigs twice a day at 0700 and 1900 with *ad libitum* access to water according to the rate of 2.0 times the maintenance requirement for ME (106 kcal of ME per kg of BW^0.75^; NRC) [14] based on the initial BW of pigs.

Fecal samples were collected 5 days after 5 days of adapta tion period, and chromic oxide and ferric oxide were used as initial and end markers, respectively. Collected excreta were frozen immediately at −20°C for the collection period, dried (60°C, 72 h) in an air-drying oven, and ground (5-mm screen, Wiley mill) for chemical analysis at the end of the trial. Urine samples were also collected daily in a plastic container with 50 mL of 10% H_2_SO_4_ to avoid evaporation of ammonia from urine, and glass wool was used as a filter to remove foreign materials. Individual collected urine was massed up to 4,000 mL with water and mixed evenly. The representative samples were collected in 50-mL conical tubes and frozen at −20°C for nitrogen retention analysis.

### Chemical analysis

Experimental diets and fecal samples were analyzed for dry matter (DM; method 934.01), CP (method 990.03), and ether extract (method 920.39 A). In addition, diets were used for analyzing crude fiber (method 978.10), ash (method 942.05), Ca (method 965.14/985.01), and P (method 965.17/985.01) by AOAC method [15]. The starch contents of the diets were determined by the polarimetric method according to the Commission Directive 1999/79/EC, and the degree of gelatinization was measured by a glucose analyzer (Model 2700, YSI, Yellow Springs, OH, USA). The chemical composition of the analyzed diets is shown in Table 2.

### Carcass characteristics

Thirty six finishing pigs (6 pigs for each treatment) were selected and slaughtered to measure the carcass weight, percentage carcass yield and backfat thickness. Carcass yield was calculated by dividing the carcass weight at the abattoir by the live weight at the farm before transport to the abattoir. Backfat thickness was measured between the 11th and 12th points located vertically with the dorsal midline.

### Gastric health

**Figure 1:**
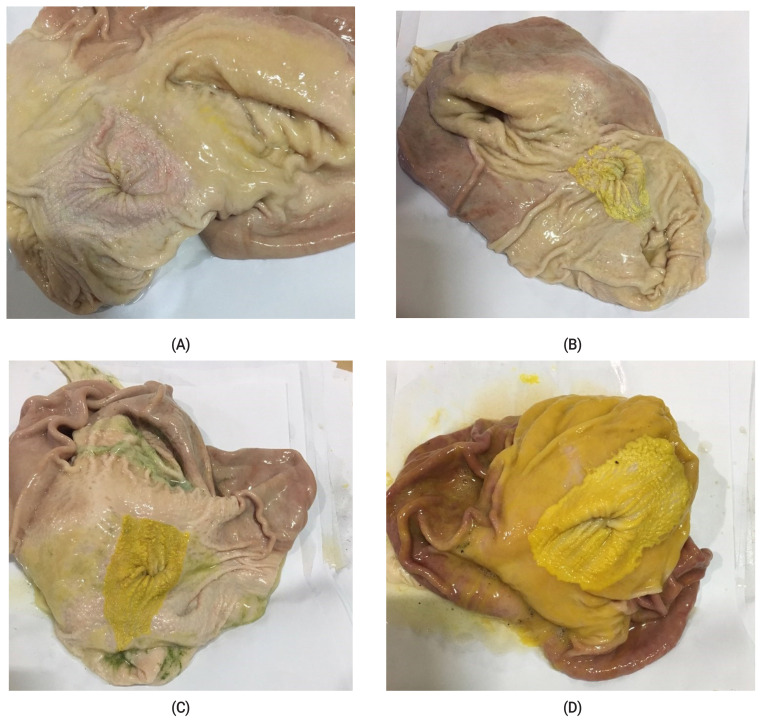
Keratinization incidence scoring standard. The (A) represents keratinization score 1 being normal or no keratinization of the esophageal region; (B) represents keratinization score 2 being keratin covering <25% of the esophageal region; (C) represents keratinization score 3 being keratin covering 25% to 75% of the esophageal region; and (D) represents keratinization score 4 being keratin covering >75% of the esophageal region.

Stomach samples were collected from individual pigs for carcass characteristics during the evisceration process and used for determining ulcer and keratinization scores by the method of De Jong [16]. Keratinization scores were assigned on a scale from 1 to 4, with 1 being normal or no keratinization of the esophageal region; 2 being keratin covering <25% of the esophageal region; 3 being keratin covering 25% to 75% of the esophageal region; and 4 being keratin covering >75% of the esophageal region. The visual criterion for the keratinization score is shown in Figure 1. Ulcer scores were also assigned on a scale from 1 to 4, with 1 being no ulcers present; 2 being ulceration affecting <25% of the esophageal region; 3 ulceration affecting 25% to 75% of the esophageal region; and 4 being ulceration affecting >75% of the esophageal region.

### Statistical analysis

All collected data were compared by least squares mean comparisons with the general linear model procedure of SAS (SAS Institute Inc., Cary, NC, USA,). As an experimental unit, individual pens were applied for analyzing performance data, whereas individual pigs were applied for analyzing nutrient digestibility, carcass characteristics, incidence of ulcers and keratinization in the stomach. The experimental unit was analyzed based on 2×3 factorial arrangements, and 2 main factors were feed form and particle size. Based on least significant-difference test, the differences were declared significant at p<0.05 or highly significant at p<0.01, and the determination of tendency for all analyses was p>0.05 and p<0.10.

## RESULTS

### Growth performance

The effect of feed form and particle size on the growth performance of growing and finishing pigs is presented in Table 3. During the whole experimental period, there was no significant difference in BW and ADG among all dietary treatments. The finishing pigs fed the mash diet had higher ADFI than those fed the pellet diet for wks 7 to 10 (p<0.05), 11 to 12 (p<0.05), and 7 to 12 (p<0.05); however, different feed forms had no effect on the ADFI of growing pigs. For the overall period, there was a tendency for improved ADFI when the pigs were fed the mash diet (p = 0.09).

The feed efficiency of pigs was consistently shown to be improved when the pigs were fed the pellet diet, regardless of the experimental period (wks 0 to 3, p<0.01; 0 to 6, p<0.01; 7 to 10, p<0.05; 11 to 12, p<0.05; 7 to 12, p<0.01; 0 to 12, p< 0.01). Decreasing particle size also improved the feed efficiency of finishing pigs at wks 7 to 10 (p<0.01), 7 to 12 (p<0.05), and 0 to 12 (p<0.05). For all parameters of the growth trial, there was no interaction between the particle size and feed form.

### Nutrient digestibility

Feeding the pellet diet improved crude fat digestibility (p< 0.01) relative to the mash diet (Table 4). However, the total tract digestibilities of DM and CP were not affected by different feed forms and particle sizes.

### Carcass characteristics

The live weight, carcass yield and backfat thickness were measured to evaluate treatment effects on carcass characteristics (Table 5). For all parameters, there was no considerable change by dietary treatments.

### Gastric health

The effects of feed form and particle size on the ulceration and keratinization scores of finishing pigs are presented in Table 6. Because of the well-managed environment, there were no pigs with ulceration problems, and dietary feed form had no significant difference on keratinization of the esophageal region. However, a tendency for an increased incidence of keratinization was observed as particle size decreased (p = 0.07). There was no considerable interaction between particle size and feed form.

## DISCUSSION

Comparing pellet and mash diets, many studies have described decreased amounts of feed waste when a pellet diet was provided to the pigs [17,18]. For feed consumption, improved intake of weanling pigs with pellet diet was observed [13], but the response was inconsistent for finishing pigs [18]. Different responses of feed intake by feed form could be induced by environmental conditions and the age of animals [19]. In a well-managed environment, animals would have maximum feed intake, and it is difficult to show treatment effects. Then, changing feed waste by different feed forms could have more effects on recorded feed intake than true feed intake. In this experiment, pigs showed high feed intake during the entire experimental period compared with the normal standard curve for feed intake, and the feed intake was improved in the mash diet, especially for the finishing phase. This result probably resulted from the reduced feed waste of the pellet diet; however, further study is needed to determine the clear reason for the increased feed intake of finishing pigs by the mash diet. Different feed intake by various particle sizes was observed in many studies, but the response was not consistent [4,20]. In this experiment, different particle sizes exerted no considerable change on feed intake, which means that there is no negative effect on feed intake if the diet is ground below 900 μm.

Generally, it is well known that the pelleting process im proves the starch digestibility of cereal grains due to the increased gelatinization degree of starch [1,2,10]. There were several findings for an improved feed conversion ratio ranging from 4% to 12% by applying a pellet diet [11,21]. In this trial, an improved G/F ratio was observed in pigs fed a pellet diet during the whole experimental period, in agreement with previous findings. Reduced feed waste by applying a pellet diet could also have an effect on improving feed efficiency, aligning with the results of feed intake. Particle size reduction had positive effects on the feed efficiency of swine in previous studies [3,22]. Ohh et al [23] reported that a fine grinding process for corn and sorghum could improve feed efficiency in the starter period, and Wondra et al [24] found that decreasing the particle size of corn resulted in an 8% improvement of feed efficiency in the growing period (47.8 kg of initial BW). In the present study, the pigs fed diets with a particle size of 900 μm showed a lower G/F ratio than those fed diets with particle sizes of 600 and 750 μm, indicating that a reduced particle size below 750 μm could improve feed efficiency in both pellet and mash diets.

Pelleting often resulted in improved ADG and feed effi ciency compared with mash diet, and it was derived from improved energy digestibility and reduced feed intake [1,2, 12,25]. Reduced particle size may improve enzyme surface reactions and increase digestibility of nutrients [2,3]. However, these responses of growth could be inconsistent due to changed digestibility by feed intake and environmental conditions [9]. In this trial, the pigs fed a pellet diet with reduced particle size showed numerically higher ADG than other treatments, with a similar trend as in previous studies, but there was no significant difference.

To evaluate the effects of dietary treatments on a total collection digestibility of growing pigs were analyzed. Several findings demonstrated that pelleting could improve digestibilities of DM, N, and energy ranging from 5% to 8% [24], and increased AID of indispensable AA by application of a pellet diet was also observed [26]. In previous studies of positive effects, the main reason for improved digestibility by pelleting was increased starch gelatinization and changed protein confirmation by the steam conditioning process; however, some findings demonstrated inconsistent results with N and AA digestibility because of different pelleting and steam conditions [27,28]. In this study, crude fat digestibility was improved by pelleting, in agreement with previous findings [10], but the digestibilities for DM and CP were not affected by dietary treatments. In the digestibility trial of this study, a restricted feeding method was applied, and the pigs were housed in a well-managed environment. Consequently, the DM and CP digestibilities of pigs fed mash and pellet diets were all over 94%, and it was difficult to differentiate treatment effects.

Increased nutrient digestibility by reduced particle size has been demonstrated consistently in previous studies [29, 30], and a popular approach for this improvement is prolonged passage rate of digesta and increased surface area for enzyme reaction. Appel [31] reported that the flowability of digesta was reduced as particle size decreased, and Jensen and Becker [10] found that reduced particle size may lead to improved energy digestibility. However, the nutrient digestibilities of DM, CP, and crude fiber were not changed by different particle sizes in this trial, which means that particle sizes between 600 and 900 μm show no difference in the digestibility of growing pigs with restricted feeding methods. In the growth trial, *ad libitum* access to feed was applied, and an improved G/F ratio was observed by reduced particle size. These different feeding programs could induce different digestibilities for various parameters, and further experiments are needed to validate the effects of the feeding program on the response of particle size.

Few studies have validated the interaction between feed form and particle size [12,24]. To improve the quality and hardness of pellets, a reduced particle size is essential; however, the standard particle size for the pelleting process was inconsistent because of different production conditions and diet compositions. In the present study, an interaction response between the two factors was not observed in all parameters, and these results suggested that the effects of pellets on growth and digestibility were not changed with particle size ranging from 600 to 900 μm.

Potter et al [18] demonstrated that carcass yield and back fat thickness after slaughter were increased when pigs were fed a pellet diet relative to those fed a mash diet, and the main reason for this change was improved energy digestibility and reduced organ weight. However, the pigs fed the pellet diet had no effect on carcass traits in this trial. In some cases, carcass yield was increased by reduced particle size because of decreased organ weight [4]. However, a significant effect of carcass characteristics by different particle sizes was not observed in this experiment, and different particle size ranges would be one of the reasons for this difference. In the study of Rojas and Stein [4], the particle size ranged from 339 to 865 μm, relatively lower than 600 μm, and this result indicated that particle size up to 600 μm had no effects on the carcass characteristics of finishing pigs.

The esophageal region is the most risky region for devel oping gastric ulcers, and an increased incidence of gastric ulcers by reduced particle size was reported by previous studies [7,8]. However, those responses could be inconsistent based on other factors, such as management methods and type of housing [32,33]. In the present study, there were no pigs with gastric ulcer problems but there was a tendency for increased keratinization score as particle size increased (p = 0.07). Development of ulcer in stomach is derived from keratinization of esophageal region. Frequent peristalsis of gut stimulates the hardening of the epithelium cells and that region of keratinization was stained by bile from small intestine. That region absorbs stains and the color is changed to yellow. Swelling and erosion the area of keratinization is the following step of ulceration [34].

For the overall period, the feed efficiency of pigs was im proved with the pellet diet and the average particle size of 600 μm. Pelleting had no effects on DM and CP digestibilities but resulted in improved crude fat digestibility relative to the mash diet. There was no considerable change in carcass characteristics by dietary treatments, but a trend for increasing incidence of keratinization of the esophageal region was observed with reduced particle size.

## Figures and Tables

**Table 1:** The formulas and chemical compositions of the growing and finishing diets

Items	Growing diet (%)	Finishing diet (%)
Ingredients (%)		
Corn	40.57	45.81
Wheat	35.00	35.00
Soybean meal	16.70	13.66
Mixed animal fat	3.84	2.30
Mono-di calcium phosphate	1.24	0.82
Limestone	0.76	0.68
Salt	0.40	0.40
L-lysine HCl (78%)	0.57	0.55
DL-methionine (99%)	0.20	0.18
L-tryptophan (99%)	0.05	0.05
L-threonine (99%)	0.23	0.22
Vitamin Mix1)	0.05	0.05
Mineral Mix2)	0.34	0.23
Choline Cl (50%)	0.05	0.05
Total	100.00	100.00
Chemical composition3)		
ME (kcal/kg)	3,300.00	3,275.00
CP (%)	15.00	14.00
Lys (%)	1.11	1.01
Met (%)	0.43	0.39
Ca (%)	0.66	0.52
Total P (%)	0.56	0.47

ME, metabolizable energy; CP, crude protein.

1) Provided per kg of diet: vitamin A, 12,000 IU; vitamin D_3_, 2,400 IU; vitamin E, 10 IU; vitamin K, 5.6 mg; vitamin B_2_, 4 mg; vitamin B_6_, 2 mg; vitamin B_12_, 40 μg; pantothenic acid, 16 mg; biotin, 100 μg; niacin, 20 mg; folic acid, 1 mg.

2) Provided per kg of diet: Fe, 65 mg; Mn, 30 mg; Zn, 30 mg; Cu, 50 mg; Se, 500 μg; I, 1.24 mg.

3) Calculated values.

**Table 2:** Proximate compositions (%) of the growing and finishing diets1)

Items	Feed form Particle size (μm)	Mash			Pellet		
	
		600	750	900	600	750	900
Growing diet							
Moisture		11.45	11.51	11.53	11.15	11.77	12.25
Crude protein		15.03	14.81	14.52	14.49	14.67	14.83
Crude fat		5.50	6.18	5.76	6.36	6.07	5.87
Crude fiber		2.61	2.43	2.38	2.62	2.88	2.50
Crude ash		4.15	4.12	4.13	4.14	4.31	4.18
Ca		0.66	0.66	0.64	0.64	0.66	0.62
Total P		0.57	0.56	0.55	0.59	0.58	0.57
Starch		48.72	48.00	48.82	49.13	47.96	47.31
Gelatinization		23.21	24.04	24.95	24.45	24.75	24.67
Finishing diet							
Moisture		11.44	11.56	11.95	12.12	13.48	12.12
Crude protein		13.81	13.98	14.10	13.56	14.05	13.56
Crude fat		4.38	4.49	4.45	4.95	4.75	4.95
Crude fiber		2.41	2.56	2.79	2.33	1.91	2.33
Crude ash		3.73	3.80	3.65	3.56	3.67	3.56
Ca		0.52	0.53	0.52	0.53	0.55	0.53
Total P		0.49	0.46	0.45	0.45	0.43	0.45
Starch		51.47	51.39	51.66	51.62	49.17	51.62
Gelatinization		21.92	21.68	23.91	29.63	29.16	29.63

1) Analyzed values.

**Table 3:** The effect of feed form and particle size on the growth performance of growing and finishing pigs

Items	Feed form		Particle size (μm)			SEM	p-value1)		
		
	Mash	Pellet	600	750	900		F	PS	F×PS
Body weight (kg)									
Initial	22.64	22.64	22.63	22.64	22.65	0.580	0.67	0.99	0.84
3 wk	37.70	38.69	38.62	38.35	37.62	1.072	0.97	0.94	0.97
6 wk	57.56	58.22	58.33	57.96	57.38	1.187	0.81	0.97	0.80
10 wk	85.58	86.37	87.62	86.30	84.01	1.356	0.91	0.62	0.82
12 wk	100.09	100.99	101.71	101.14	98.79	1.411	0.98	0.73	0.93
ADG (g)									
0–3 wk	717	765	762	749	713	25.2	0.59	0.70	0.86
4–6 wk	946	930	939	934	941	14.0	0.24	0.93	0.17
0–6 wk	831	847	850	841	827	18.7	0.63	0.58	0.36
7–10 wk	1,001	999	1,039	1,013	952	13.0	0.86	0.03	0.93
11–12 wk	1,037	1,044	1,007	1,060	1,056	19.9	0.66	0.53	0.40
7–12 wk	1,013	1,015	1,027	1,029	986	10.0	0.66	0.17	0.75
0–12 wk	922	933	942	934	907	10.8	0.81	0.42	0.95
ADFI (g)									
0–3 wk	1,345	1,319	1,344	1,338	1,314	43.6	0.52	0.97	0.90
4–6 wk	1,936	1,830	1,893	1,854	1,903	42.8	0.10	0.84	0.53
0–6 wk	1,640	1,574	1,618	1,596	1,608	41.8	0.25	0.98	0.74
7–10 wk	2,625	2,491	2,523	2,552	2,600	33.0	0.04	0.48	0.19
11–12 wk	2,576	2,444	2,480	2,576	2,475	36.3	0.04	0.74	0.51
7–12 wk	2,609	2,469	2,508	2,550	2,559	31.5	0.03	0.67	0.52
0–12 wk	2,125	2,022	2,064	2,073	2,084	34.3	0.09	0.93	0.66
G:F ratio									
0–3 wk	0.533	0.581	0.568	0.561	0.542	0.071	<0.01	0.11	0.90
4–6 wk	0.493	0.517	0.508	0.510	0.499	0.080	0.13	0.83	0.53
0–6 wk	0.510	0.544	0.533	0.532	0.517	0.058	<0.01	0.30	0.68
7–10 wk	0.383	0.403	0.413	0.398	0.368	0.063	0.04	<0.01	0.28
11–12 wk	0.402	0.431	0.407	0.414	0.429	0.082	0.03	0.66	0.30
7–12 wk	0.389	0.412	0.411	0.405	0.387	0.050	<0.01	0.04	0.16
0–12 wk	0.435	0.463	0.459	0.453	0.437	0.043	<0.01	0.01	0.28

SEM, standard error of mean; ADG, average daily gain; ADFI, average daily feed intake; G:F ratio, gain-to-feed ratio.

1) F, feed form; PS, particle size.

**Table 4:** The effect of feed form and particle size on the total collection digestibility of growing pigs1)

Items	Feed form		Particle size (μm)			SEM	p-value2)		
		
	Mash	Pellet	600	750	900		F	PS	F×PS
Nutrient digestibility (%)									
Dry matter	95.00	95.14	94.91	95.26	95.04	0.09	0.42	0.25	0.21
Crude protein	94.52	94.16	94.21	94.56	94.26	0.14	0.19	0.53	0.32
Crude fat	91.57	93.70	92.55	92.99	92.37	0.31	<0.01	0.38	0.29
Nitrogen retention (g/d)									
N intake	29.15	29.15	29.15	29.15	29.15	-	-	-	-
Fecal N	1.61	1.69	1.66	1.57	1.71	0.04	0.28	0.37	0.16
Urinary N	1.22	1.17	1.20	1.23	1.14	0.19	0.16	0.14	0.63
N retention3)	26.32	26.29	26.28	26.34	26.29	0.04	0.68	0.81	0.26

SEM, standard error of the mean.

1) A total of 24 growing pigs were fed an average initial body weight of 33.65±0.372 kg.

2) F, feed form; PS, particle size.

3) N retention = N intake – fecal N – urinary N.

**Table 5:** The effect of feed form and particle size on carcass characteristics of finishing pigs

Item	Feed form		Particle size (μm)			SEM	p-value1)		
		
	Mash	Pellet	600	750	900		F	PS	F×PS
Live weight (kg)	111.2	112.0	111.8	111.4	111.4	1.30	0.77	0.96	0.67
Carcass yield (%)	77.0	76.8	77.0	76.8	76.9	0.05	0.22	0.69	0.68
Back fat P_2_ (mm)	23.5	24.3	23.5	24.2	23.8	0.94	0.64	0.98	0.38

SEM, standard error of mean.

1) F, feed form; PS, particle size.

**Table 6:** The effect of feed form and particle size on ulceration and keratinization of finishing pigs

Items	Feed form		Particle size (μm)			SEM	p-value1)		
		
	Mash	Pellet	600	750	900		F	PS	F×PS
Keratinizaition2)	1.83	1.72	2.25	1.75	1.34	0.170	0.66	0.07	0.23
Ulceration3)	0.0	0.0	0.0	0.0	0.0	-	-	-	-

SEM, standard error of mean.

1) F, feed form; PS, particle size.

2) 1 being normal or no keratinization of the esophageal region; 2 being keratin covering <25% of the esophageal region; 3 being keratin covering 25% to 75% of the esophageal region; and 4 being keratin covering >75% of the esophageal region.

3) Ulcer scores were also assigned on a scale from 1 to 4 with 1 being no ulcers present; 2 being ulceration affecting <25% of the esophageal region; 3 ulceration affecting 25% to 75% of the esophageal region; and 4 being ulceration affecting >75% of the esophageal region.
