# Corrigendum

**DOI:** 10.5713/ab.20.0777C

**Published:** 2026-02-24

The following was corrected in **Anim Biosci Vol 34, No 6 : 1061–1069**.

**Previous author list:**

Yun Yeong Jo^1^, **Myung Jae Choi**^2^, Woo Lim Chung^2^, Jin Su Hong^2^, Jong Seon Lim^1^, and Yoo Yong Kim^2,^*

**Updated author list:**

Yun Yeong Jo^1^, **Myeong Jae Choi**^2^, Woo Lim Chung^2^, Jin Su Hong^2^, Jong Seon Lim^1^, and Yoo Yong Kim^2,^*

